# Angiossarcoma Pericárdico Primário Complicado por Pericardite Constritiva

**DOI:** 10.36660/abc.20240683

**Published:** 2025-04-25

**Authors:** Guilherme Augusto Reissig Pereira, Maiane Maria Pauletto, Anna Paula Tscheika, Fabrício Michalski Velho, Luciano Folador

**Affiliations:** 1 Hospital de Clínicas de Porto Alegre Porto Alegre RS Brasil Hospital de Clínicas de Porto Alegre, Porto Alegre, RS – Brasil; 2 Hospital São Lucas da PUCRS Porto Alegre RS Brasil Hospital São Lucas da PUCRS, Porto Alegre, RS – Brasil

**Keywords:** Tumor Cardíaco, Angiossarcoma Cardíaco Primário, Angiossarcoma, Neoplasias Cardíacas, Pericardite Constritiva

## Introdução

Tumores cardíacos malignos secundários são metástases de tumores primários e 22-132 vezes mais comuns que tumores cardíacos primários (TCP).^1 - 3^ Os TCPs são raros, com uma prevalência na autópsia estimada de 0,001-0,03%,^4^ e majoritariamente benignos.^5 , 6^

Entre os TCPs, o angiossarcoma cardíaco é um subtipo raro de sarcoma de tecido mole, e o tumor primário maligno mais comum do coração.^5^ Os angiossarcomas consistem em canais vasculares de formato irregular, revestidos por células epiteliais anaplásticas, com grandes áreas de necrose e hemorragia.^1^ O angiossarcoma cardíaco é um tumor agressivo com uma alta taxa de recorrência local de disseminação sistêmica.^7^ Sem ressecção cirúrgica, a sobrevida global dos pacientes é de 3,8 (DP 2,5) meses.^1^ O tumor cresce e se infiltra rapidamente, o que pode levar a manifestações clínicas incluindo ruptura do miocárdio, derrame pericárdico, tamponamento cardíaco e pericardite constritiva (PC).^5^

Em países de alta renda, a tuberculose é a principal causa de PC, enquanto em outras partes do mundo, causas virais ou idiopáticas continuam a etiologia mais comum. Em uma minoria dos casos, uma doença maligna cardíaca é identificada como a etiologia de PC.^8^

Relatamos um caso de angiossarcoma pericárdico complicado por PC e apresentamos uma revisão da literatura relevante.

## Descrição

Um homem de 46 anos, branco, com hipotireoidismo, diabetes tipo 1, vitiligo, e tumor de células germinativas não-seminomatosas aos 24 anos de idade, apresentou-se com dor retrosternal, dispneia aos esforços moderados, e sudorese noturna por um mês. O exame físico revelou taquicardia, taquipneia, pressão arterial normal, pressão venosa jugular aumentada, sons cardíacos normais, e sons respiratórios vesiculares diminuídos em base pulmonar.

Exames laboratoriais mostraram hemoglobina de 9,3 g/dL, contagem de leucócitos de 9500 leucócitos/mm^3^, neutrófilos 72%, plaquetas 624 000/mm^3^, proteína C reativa 23 mg/dL, creatinina 0,9 mg/dL, troponina 22 ng/mL, porção N-terminal do pró-hormônio do peptídeo natriurético do tipo B (NT-pro-BNP) 2750 pg/mL, anticorpo antinuclear negativo, fator reumatoide negativo, e concentrações normais de complementos C3 e C4. O eletrocardiograma mostrou taquicardia sinusal, baixa voltagem, e anormalidades de repolarização não específicas. Ecocardiograma transtorácico (ETT) bidimensional revelou grane derrame pericárdico.

O paciente foi submetido a uma pericardiocentese, com remoção de 650 mL de líquido. Houve recorrência de derrame pericárdico e tamponamento cardíaco dentro de um curto período e, por isso, realizou-se uma janela pericárdica, com drenagem de 900mL de líquido com sangue. A análise do líquido mostrou teste negativo para adenosina desaminase, culturas negativas, e a citologia deu negativa para células malignas. Uma biópsia do pericárdio também não mostrou evidência de malignidade.

**Figura 1:**
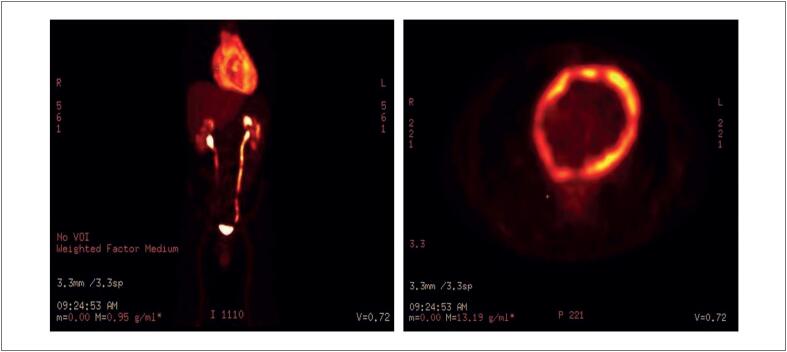
Tomografia por emissão de pósitrons/Tomografia Computadorizada usando fluorodesoxiglicose (^18^F-FDG-PET/CT) revela atividade hipermetabólica do pericárdio, com um valor de captação padronizado máximo (SUVmax) de 12,8 g/mL.

**Figura 2:**
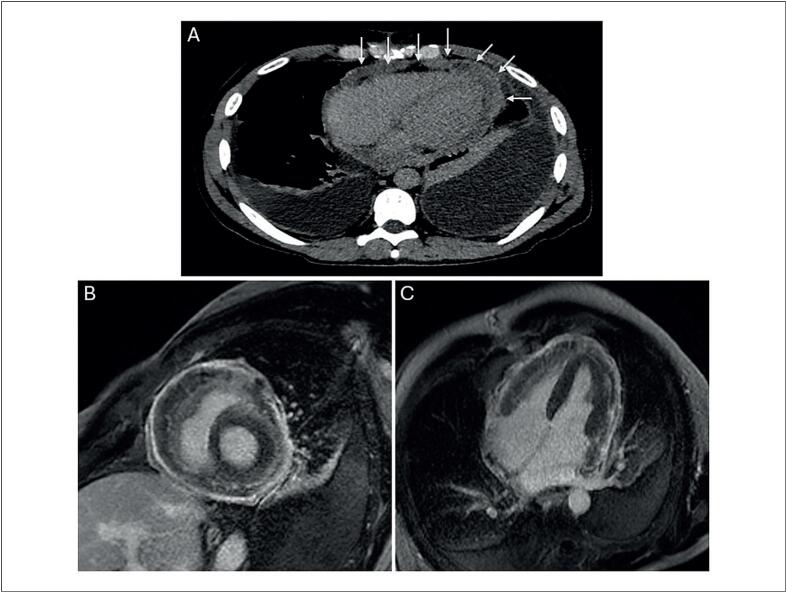
Tomografia computadorizada cardíaca pós-contraste (A), ressonância magnética cardíaca com realce tardio de gadolínio: corte eixo curto (B) e corte quatro câmaras (C) mostrando realce difuso e espessamento nodular (setas brancas) do pericárdio com derrame pericárdico leve.

**Figura 3:**
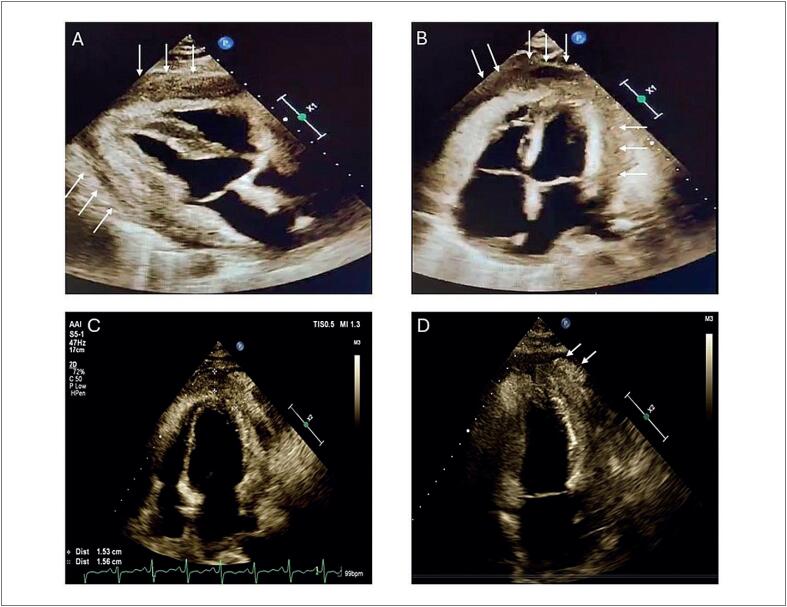
Ecocardiograma Transtorácico (ETT) corte eixo longo (A) e corte apical quatro câmaras (B) mostrando uma massa pericárdica ecogênica (setas brancas) ao redor dos ventrículos, e se estendendo até próximo às válvulas atrioventriculares; ETT em corte quatro câmaras (C) e duas câmaras (D) revelando espessamento do pericárdio anterior e apical de 15mm, com nodulação (setas brancas).

**Figura 4:**
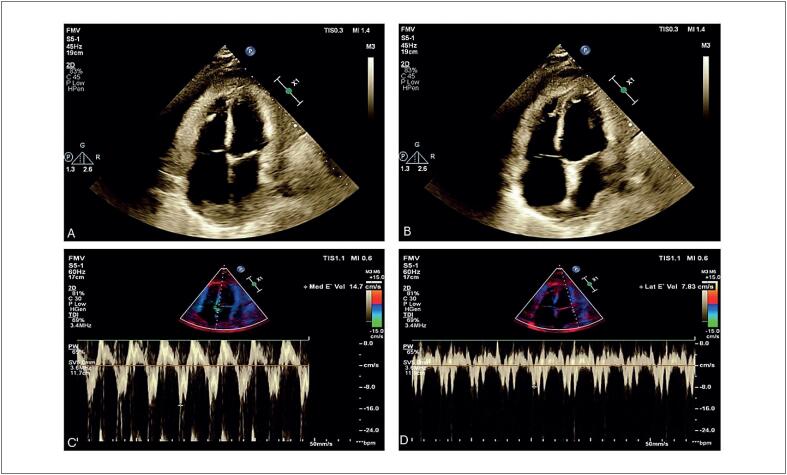
Sinais ecocardiográficos de pericardite constritiva; Ecocardiograma Transtorácico (ETT) em corte quatro câmaras mostrando movimento anômalo do septo interventricular (" *septal bounce* ") (A-B); Doppler tecidual do anel mitral septal (C) revela uma velocidade anular mitral diastólica precoce (e’) medial de 14,7 cm/s, a qual é mais alta que a velocidade lateral e’ de 7,8 cm/s (D), caracterizando annulus reversus.

A tomografia por emissão de pósitrons/Tomografia Computadorizada usando fluorodesoxiglicose (^18^F-FDG-PET/TC) revelou espessamento difuso dos folhetos pericárdicos com formato irregular, realce com contraste, e atividade hipermetabólica com um valor de captação padronizado máximo (SUVmax, *standardized uptake value* ) de 12,8 ( Figura 1 ). Em seguida, o paciente apresentou aumento da pressão jugular venosa, edema nos tornozelos, e dispneia progressiva. A ausculta pulmonar revelou ausência de sons respiratórios vesiculares na metade inferior de ambos os pulmões. A TC cardíaca mostrou grande derrame pleural bilateral, espessamento septal interlobular, e significativo espessamento nodular dos folhetos pericárdicos ( Figura 2A ). O exame de Ressonância Magnética Cardíaca (RMC) confirmou extenso realce por gadolínio no pericárdio ( Figuras 2B-C ). Um novo ETT mostrou derrame pericárdico leve e presença de uma massa pericárdica ecogênica ao redor dos ventrículos, estendendo-se próximo às válvulas atrioventriculares ( Figuras 3A e B e vídeos 1 - 2 ). O ETT também revelou a presença de "annulus reversus" e movimento atípico do septo interventricular, sugestivo de PC ( Figura 4 ). O paciente recebeu furosemida e foi submetido à drenagem pleural bilateral e pericardiectomia. Análise patológica do pericárdio revelou positividade imuno-histoquímica para CD31, ERG, e D2-40, e um índice de proliferação Ki-67 de 90%, consistente com angiossarcoma pericárdico.

O tratamento foi iniciado com paclitaxel 80 mg/m^2^, que levou à melhora clínica e remoção dos drenos do tórax e descontinuação do oxigênio. O paciente recebeu alta com uma r prescrição de furosemida e bisoprolol.

**Figura 5:**
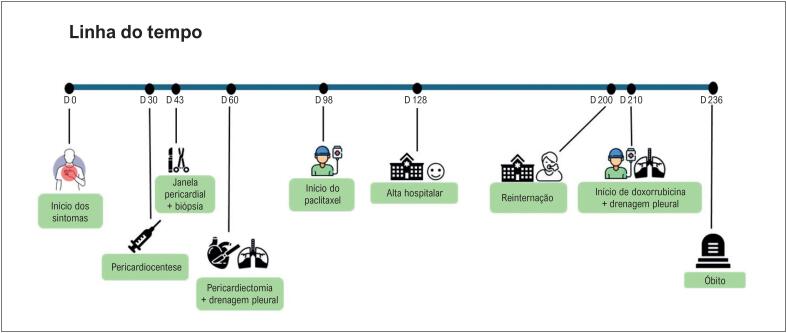
Linha do tempo do caso apresentado.

Dois meses e meio depois, o paciente foi readmitido no hospital por dispneia a mínimos esforços. O exame de imagem revelou um derrame pleural loculado e um nódulo no lóbulo inferior do pulmão direito, sugestivo de metástase. O ETT mostrou sinais de PC e espessamento do pericárdio a 15mm ( Figuras 3C-D e vídeos 3 - 4 ). Devido ao alto grau de invasão, dificuldade em se definir o plano de clivagem, e à condição clínica do paciente, não se decidiu por cirurgia. A dose de furosemida foi aumentada, realizou-se drenagem pleural, um ciclo por mês. Infelizmente, o paciente continuou a apresentar piora clínica e disfunção ventilatória. O paciente passou a ser mantido em cuidados paliativos, e foi a óbito oito meses após o início dos sintomas ( Figura 5 ).

## Discussão

O angiossarcoma cardíaco afeta preferencialmente homens jovens, com um pico de incidência na quarta década de vida.^1^ Sua origem é atrial em 75% dos casos, tipicamente preenche essa câmara e então infiltra no pericárdio, válvula tricúspide, ventrículo direito, e artéria coronária direita. Em 47% a 89% dos casos, metástases ocorrem mais comumente nos pulmões (22-55%), mas também no fígado, ossos, cólon e cérebro.^1 , 9^ Os sintomas iniciais são inespecíficos,^4 , 5^ e as manifestações mais comuns são dispneia (50-80%), derrame pericárdico (29-56%), e dor torácica (10-39%).^9^ Sintomas constitucionais também podem estar presentes, incluindo perda de peso e fadiga relacionada à anemia.^7^

A incidência de TCPs aumentou na última década.^6^ Parte desse aumento pode ser atribuída a um melhor acesso e avanços nas modalidades de imagens.^1^ O ETT é geralmente a primeira modalidade de imagem utilizada, que permite a avaliação do tamanho, da localização e da mobilidade do tumor, e do envolvimento do pericárdio. A RMC é útil por possibilitar uma avaliação multiplanar da massa e seu potencial envolvimento com as câmaras cardíacas e o pericárdio.^1^ Similar à RMC, a TC cardíaca fornece informações sobre as margens das lesões e sua relação com estrutura extracardíacas.^1^

O escaneamento com ^18^F-FDG-PET/TC é útil por investigar tumores cardíacos avaliando-se sua atividade metabólica, que se baseia tipicamente na avaliação da média do SUVmax. Em um estudo com 20 pacientes que foram submetidos ao escaneamento com ^18^F-FDG-PET/TC, a técnica demonstrou uma sensibilidade de 100% e uma especificidade de 92% para diferenciar massas cardíacas malignas de massas cardíacas benignas. A SUVmax foi mais alta nos casos de malignidade (13,2 ± 6,2 g/mL) que nos casos não malignos (2,3 ± 1,2 g/mL) (p=0,0004).^10^ A SUVmax observada nos casos malignos é similar à encontrada em nosso caso: 12,8 g/mL.

A raridade do angiossarcoma cardíaco torna difícil a padronização dos algoritmos de tratamento.^7^ A maioria dos estudos são séries de casos retrospectivos ou relatos de casos envolvendo populações heterogêneas.^5^ Os melhores desfechos de longo prazo são obtidos com ressecção cirúrgica com margens negativas, mas isso é geralmente impedido pela presença de metástase e proximidade do tumor aos condutos vasculares. Quando a ressecção completa é possível, os pacientes geralmente sofrem recorrência local. A quimioterapia neoadjuvante, combinada com a remoção do tumor pode promover uma vantagem de sobrevida pela redução do tamanho do tumor.^1 , 7^

O angiossarcoma cardíaco metastático é tratado com quimioterapia sistêmica. Antraciclinas estão associadas com uma taxa de resposta de 16-27% e uma sobrevida mediana de até 12 meses.^4^ O paclitaxel também é comumente utilizado devido às suas propriedades antiangiogênicas. No estudo ANGIOTAX, o paclitaxel foi associado a uma sobrevida mediana de oito meses nos pacientes com angiossarcoma não ressecável.^11 - 13^ O interesse na terapia anti-Fator de Crescimento Endotelial Vascular (VEGF) levou a ensaios clínicos de fase I/II com bevacizumab, porém, sua adição à quimioterapia não melhorou significativamente a sobrevida livre de progressão.^4 , 14^

A doença cardíaca maligna é uma etiologia rara de PC. A PC é crônica e progressiva na maioria dos pacientes, que tipicamente apresentam sinais de insuficiência cardíaca direita que potencialmente causa dispneia por derrame pleural.^15^ O ETT permite a detecção dessa condição. O pericárdio constritivo limita o volume cardíaco. Durante a inspiração, o ventrículo direito não consegue se expandir para acomodar o retorno venoso aumentado e, em vez disso, seu volume aumenta e invade o espaço ventricular esquerdo devido a um desvio no septo ventricular. Por outro lado, durante a expiração, o septo retorna em direção ao ventrículo direito. Esse fenômeno, conhecido como o movimento anômalo do septo interventricular conhecido como " *septal bounce* ", tem uma sensibilidade de 93% para detectar PC. Outro importante achado ecocardiográfico é *annulus reversus* , em que a velocidade e’ mitral medial precoce (e’) é mais alta que a velocidade e’ lateral.^15 , 16^

Embora a terapia diurética reduza sintomas, ela não modifica o curso natural da PC. O único tratamento definitivo é a pericardiectomia cirúrgica, apesar da alta mortalidade perioperatória.^15^ Em um centro de grande volume, 89 pacientes com PC foram submetidos à pericardiectomia, com uma taxa de mortalidade perioperatória de 7%.^17^

Em resumo, apresentamos um caso de angiossarcoma cardíaco complicado por PC, com um desfecho clínico desfavorável apesar da pericardiectomia e da quimioterapia. O desafio em tratar esse tumor está na sua raridade, os estágios tipicamente avançados no momento do diagnóstico, seu curso agressivo com alto potencial metastático, e o risco de recorrência. Quando viável, a ressecção cirúrgica completa é a melhor opção paliativa, mas ela raramente oferece uma chance de cura.

## *Material suplementar

Para assistir ao vídeo suplementar 1, por favor, clique aqui .

Video 1

Para assistir ao vídeo suplementar 2, por favor, clique aqui .

Video 2

Para assistir ao vídeo suplementar 3, por favor, clique aqui .

Video 3

Para assistir ao vídeo suplementar 4, por favor, clique aqui .

Video 4
