# Supplementary material for: Angiossarcoma Pericárdico Primário Complicado por Pericardite Constritiva
Source: Arq Bras Cardiol. 2025 Apr 25;122(3):e20240683. [Article in Portuguese] doi: 10.36660/abc.20240683 (PMC12094290; doi:10.36660/abc.20240683)
Supplement: Translation

# Translation: Primary Pericardial Angiosarcoma Complicated by Constrictive Pericarditis

**DOI:** 10.36660/abc.20240683i

**Authors:** Guilherme Augusto Reissig Pereira, Maiane Maria Pauletto, Anna Paula Tscheika, Fabrício Michalski Velho, Luciano Folador

**Affiliations:** 1 Porto Alegre RS Brazil Hospital de Clínicas de Porto Alegre, Porto Alegre, RS – Brazil; 2 Porto Alegre RS Brazil Hospital São Lucas da PUCRS, Porto Alegre, RS – Brazil

**Keywords:** Cardiac Tumor, Primary Cardiac Angiosarcoma, Angiosarcoma, Heart Neoplasms, Constrictive Pericarditis

## Introduction

Secondary malignant cardiac tumors are metastases from primary tumors and 22 to 132 times more common than primary cardiac tumors (PCTs).^1 - 3^PCTs are rare, with an estimated autopsy prevalence of 0.001-0.03%,^4^and mostly (90%) benign.^5 , 6^

Among PCTs, cardiac angiosarcoma is a rare subtype of soft tissue sarcoma and the most common primary malignant tumor of the heart.^5^Angiosarcomas consist of irregularly shaped vascular channels lined by anaplastic epithelial cells with substantial areas of necrosis and hemorrhage.^1^Cardiac angiosarcoma is an aggressive tumor with a high rate of local recurrence and systemic dissemination.^7^Without surgical resection, it has a mean overall survival of 3.8 (SD 2.5) months.^1^The rapid infiltrating growth of this tumor can lead to emergency clinical presentations including myocardial rupture, pericardial effusions, cardiac tamponade, and constrictive pericarditis (CP).^5^

In high-income countries, tuberculosis is the leading cause of CP, whereas in other parts of the world, idiopathic or viral causes remain the most common etiology. In a minority of cases, cardiac malignancy is identified as the etiology of CP.^8^

We report a case of pericardial angiosarcoma complicated by CP and provide a review of the relevant literature.

## Description

A 46-year-old white man with hypothyroidism, type 1 diabetes, vitiligo, and non-seminomatous germ cell tumor at age 24 presented with retrosternal pain, dyspnea on moderate exertion, and night sweats for one month. Physical examination showed tachycardia, tachypnea, normal blood pressure, elevated jugular venous pressure, normal heart sounds, and diminished vesicular breath sounds at the lung bases.

Laboratory tests revealed hemoglobin of 9.3 g/dL, leukocyte count 9500/mm^3^, neutrophils 72%, platelets 624,000/mm^3^, C-reactive protein 23 mg/dL, creatinine 0.9 mg/dL, troponin 22 ng/mL, N-terminal pro-B-type natriuretic peptide (NT-pro-BNP) 2750 pg/mL, negative antinuclear antibody, negative rheumatoid factor, and normal C3 and C4 complement levels. Electrocardiogram showed sinus tachycardia, low voltage, and nonspecific repolarization abnormalities. Two-dimensional transthoracic echocardiography (TTE) revealed a large pericardial effusion.

The patient underwent pericardiocentesis with removal of 650 mL of fluid. He experienced recurrence of pericardial effusion and cardiac tamponade within a short period of time, so a pericardial window was performed, and 900 mL of bloody fluid was drained. Fluid analysis showed a negative test for adenosine deaminase, negative cultures, and cytology was negative for malignancy. A pericardial biopsy also showed no evidence of malignancy.

Positron emission tomography/computed tomography using fluorodeoxyglucose (^18^F-FDG-PET/CT) revealed diffuse thickening of pericardial leaflets with an irregular shape, contrast enhancement, and hypermetabolic activity with a maximum standardized uptake value (SUVmax) of 12.8 g/mL ( Figure 1 ). The patient subsequently developed elevated jugular venous pressure, ascites, ankle swelling, and progressive dyspnea. Lung auscultation revealed absent vesicular breath sounds over the lower half of both lungs. Cardiac CT showed large bilateral pleural effusion, interlobular septal thickening, and significant nodular thickening of pericardial leaflets ( Figure 2A ). Cardiac magnetic resonance imaging (MRI) confirmed extensive late gadolinium enhancement in the pericardium ( Figure 2B-C ). A new TTE showed mild pericardial effusion and the presence of an echogenic pericardial mass surrounding the ventricles and extending close to the atrioventricular valves ( Figures 3A-B and videos 1 - 2 ). The TTE also revealed annulus reversus and atypical movement of the interventricular septum suggestive of CP ( Figure 4 ). The patient received furosemide and underwent bilateral pleural drainage and pericardiectomy. Pathologic examination of the pericardium revealed immunohistochemical positivity for CD31, ERG, and D2-40 and a Ki-67 proliferation index of 90% consistent with pericardial angiosarcoma.

**Figure 1:** Positron emission tomography/computed tomography using fluorodeoxyglucose (^18^F-FDG-PET/CT) reveals hypermetabolic activity of the pericardium with a maximum standardized uptake value (SUVmax) of 12.8 g/mL.

**Figure 2:** Post-contrast cardiac computed tomography (A) and post-gadolinium cardiac magnetic resonance imaging in short-axis view (B) and four-chamber view (C) shows diffuse enhancement and nodular thickening (white arrows) of the pericardium with mild pericardial effusion.

**Figure 3:** Transthoracic echocardiography (TTE) in long-axis view (A) and apical 4-chamber view (B) shows an echogenic pericardial mass (white arrows) surrounding the ventricles and extending close to the atrioventricular valves; TTE in 4-chamber view (C) and 2-chamber view (D) reveals thickening of the apical and anterior pericardium to 15 mm, with nodulation (white arrows).

**Figure 4:** Echocardiographic signs of constrictive pericarditis. TTE in the 4-chamber view shows septal bounce (A-B), an atypical movement of the interventricular septum. Tissue Doppler of the septal mitral annulus (C) reveals an early medial diastolic mitral annular velocity (e’) of 14.7 cm/s, which is higher than the lateral e’ velocity of 7.8 cm/s (D), characterizing annulus reversus.

Treatment was initiated with weekly paclitaxel 80 mg/m^2^, which led to clinical improvement allowing for the removal of chest drains and discontinuation of oxygen. The patient was discharged with a prescription for furosemide and bisoprolol.

Two and a half months later, the patient was readmitted for dyspnea on minimal exertion. Imaging revealed a large loculated pleural effusion and a nodule in the lower lobe of the right lung, suggestive of metastasis. TTE showed signs of CP again and thickening of the pericardium to 15 mm ( Figures 3C-D and videos 3 - 4 ). Due to high-grade invasion, difficulty in defining the surgical cleavage plane, and the patient's poor clinical condition, surgery was not pursued. The furosemide dose was increased, pleural drainage was performed, and second-line chemotherapy was initiated with liposomal doxorubicin 40 mg/m², one cycle per month. Unfortunately, the patient continued to experience clinical deterioration, developed ventilatory dysfunction, transitioned to palliative care, and subsequently died eight months after the onset of symptoms ( Figure 5 ).

**Figure 5:** Timeline of the case reported.

## Discussion

Cardiac angiosarcoma preferentially affects young men, with a peak incidence in the fourth decade of life.^1^It is of right atrial origin in 75% of cases, typically fills this chamber and then infiltrates into the pericardium, tricuspid valve, right ventricle, and right coronary artery. In 47% to 89% of cases, metastases develop most commonly in the lungs (22-55%) but also to the liver, bone, colon, and brain.^1 , 9^Initial symptoms are nonspecific,^4 , 5^and the most common manifestations are dyspnea (50-80%), pericardial effusion (29-56%), and chest pain (10-39%).^9^Constitutional symptoms may also be present, such as weight loss and anemia-related fatigue.^7^

The incidence of PCTs has increased over the past decade.^6^Part of this increase may be attributed to improved access and advances in imaging modalities.^1^TTE is often the first imaging modality used and allows for the assessment of tumor size, location, mobility, and pericardial involvement. Cardiac MRI is useful because it provides a multiplanar evaluation of the mass and its potential involvement with the cardiac chambers and pericardium.^1^Similar to cardiac MRI, cardiac CT provides information on lesion margins and their relationship to extracardiac structures.^1^

^18^F-FDG-PET scanning is useful for investigating cardiac tumors by evaluating their metabolic activity, which is typically based on SUVmax evaluation.^1^In a study of 20 patients who underwent to^18^F-FDG-PET scanning, the technique demonstrated a sensitivity of 100% and specificity of 92% for differentiating malignant from benign cardiac masses. The mean SUVmax was higher in malignant cases (13.2 ± 6.2 g/mL) than in nonmalignant cases (2.3 ± 1.2 g/mL) (p=0.0004).^10^The SUVmax found in malignant cases is similar to that found in our case: 12.8 g/mL.

The rarity of cardiac angiosarcoma has made it difficult to standardize treatment algorithms.^7^Most studies are retrospective case series or case reports involving heterogeneous populations.^5^The best long-term outcomes are obtained with surgical resection with negative margins, but this is often precluded by metastatic spread and close proximity of the tumor to vital vascular conduits. When complete resection is possible, patients commonly experience local recurrence. Neoadjuvant chemotherapy combined with surgical debulking may provide a survival advantage by reducing tumor size.^1 , 7^

Metastatic cardiac angiosarcoma is managed with systemic chemotherapy. Anthracyclines are associated with a response rate of 16-27% and a median overall survival of up to 12 months.^4^Paclitaxel is also commonly used due to its anti-angiogenic properties. In the ANGIOTAX study, paclitaxel was associated with a median overall survival of 8 months in patients with unresectable angiosarcoma.^11 - 13^Interest in anti-vascular endothelial growth factor (VEGF) therapy led to phase I/II trials with bevacizumab, but its addition to chemotherapy did not significantly improve progression-free survival.^4 , 14^

Cardiac malignancy is a rare etiology of CP. CP is chronic and progressive in most patients, who typically present with signs of right heart failure potentially causing dyspnea due to pleural effusion.^15^TTE allows for the detection of this condition. The constrictive pericardium limits cardiac volume. During inspiration, the right ventricle cannot expand to accommodate increased venous return; instead, its volume increases by invading into the left ventricular space due to a ventricular septal shift. Conversely, during expiration, the septum shifts back towards the right. This phenomenon, known as septal bounce, has a sensitivity of 93% for detecting CP. Another important echocardiographic finding is annulus reversus, where the medial early diastolic mitral annular velocity (e’) is higher than the lateral e’ velocity.^15 , 16^

Diuretic therapy mitigates symptoms, but it does not change the natural course of CP. The only definitive treatment is surgical pericardiectomy, despite the high perioperative mortality.^15^In a large-volume center, 89 patients with CP underwent pericardiectomy with a perioperative mortality rate of 7%.^17^

In summary, we presented a case of cardiac angiosarcoma complicated by CP with an unfavorable clinical outcome despite pericardiectomy and chemotherapy. The challenge in treating this tumor lies in its rarity, the commonly advanced stage at diagnosis, its aggressive course with high metastatic potential, and the risk of recurrence. Complete surgical resection, when feasible, is the best option for palliation, but it rarely offers a chance for cure.

## *Supplemental Materials

See the Supplemental Video 1, please click here .

Video 1

See the Supplemental Video 2, please click here .

Video 2

See the Supplemental Video 3, please click here .

Video 3

See the Supplemental Video 4, please click here .

Video 4
